# Slope Entropy Normalisation by Means of Analytical and Heuristic Reference Values

**DOI:** 10.3390/e25010066

**Published:** 2022-12-30

**Authors:** David Cuesta-Frau, Mahdy Kouka, Javier Silvestre-Blanes, Víctor Sempere-Payá

**Affiliations:** 1 Technological Institute of Informatics (ITI), Universitat Politècnica de València, Alcoi Campus, 03801 Alcoi, Spain; 2 Department of System Informatics and Computers, Universitat Politècnica de València, 46022 Valencia, Spain

**Keywords:** slope entropy, time series classification, entropy normalisation, maximum entropy, minimum entropy

## Abstract

Slope Entropy (SlpEn) is a very recently proposed entropy calculation method. It is based on the differences between consecutive values in a time series and two new input thresholds to assign a symbol to each resulting difference interval. As the histogram normalisation value, SlpEn uses the actual number of unique patterns found instead of the theoretically expected value. This maximises the information captured by the method but, as a consequence, SlpEn results do not usually fall within the classical [0,1] interval. Although this interval is not necessary at all for time series classification purposes, it is a convenient and common reference framework when entropy analyses take place. This paper describes a method to keep SlpEn results within this interval, and improves the interpretability and comparability of this measure in a similar way as for other methods. It is based on a max–min normalisation scheme, described in two steps. First, an analytic normalisation is proposed using known but very conservative bounds. Afterwards, these bounds are refined using heuristics about the behaviour of the number of patterns found in deterministic and random time series. The results confirm the suitability of the approach proposed, using a mixture of the two methods.

## 1. Introduction

Entropy–related features have been used extensively for time series classification purposes with great results. They have been applied in many scientific and technical domains, although medicine is probably the most exploited field, with outstanding results in healthy–ill subjects classification and early diagnosis tasks [1].

The recently proposed time series entropy measure termed Slope Entropy (SlpEn) [2] is able to achieve high classification accuracy using a diverse set of records [2,3,4]. It has also been already implemented in scientific software tools despite its short life, such as in EntropyHub (https://github.com/MattWillFlood/EntropyHub.jl, accessed on 29 December 2022) and CEPS, Complexity and Entropy in Physiological Signals [5].

However, more steps have to be taken towards a complete optimisation of this measure, including the refinement of the initial algorithm. This way, the method will become more convenient to use, more robust, and with enhanced generalisation capabilities, as was the case for older entropy methods in the past, an ongoing and dynamic process.

For example, an already classical method, Approximate Entropy (ApEn) [6], has been quite extensively studied and characterised to provide guidelines regarding the input parameter values [7], its behaviour depending on the length of the time series [8], its statistical properties [9], its robustness against noise [10], its sensitivity to missing data [11], and its generic properties [12], among others.

The same applies to a more recent method, Permutation Entropy (PE) [13]. Generic optimizations [14], generic recommendations [15], how to use non–uniform embedding [16], the influence of time series length [17], how to improve its robustness against ties [18], specific recommendations for parameter selection [19], how to exploit the information related to forbidden ordinal patterns [20], and the real influence of ties in classification tasks [21], are examples of the multiple characterisation studies applied to PE.

Most of these methods are based on computing the relative frequency of some direct or derived specific time series features or symbolic patterns, and applying the computation of, among others, the Shannon entropy to the resulting histogram [22]. Therefore, the relative frequency estimated values are always statistically bounded so that their sum equals 1, or at least a finite constant value known in advance such as log${}_{2}m!$ [15] (being *m* the embedded dimension as defined later).

Nevertheless, other recent studies have demonstrated that the total number of symbolic patterns theoretically possible or expected, and the actual number found or extracted even from an infinite length time series, could greatly differ due to the so–called forbidden patterns [23]. Since this number of forbidden patterns is strongly related to the determinism degree of the time series under analysis [24,25], other works proposed to use the real number of patterns found instead of the theoretical one to compute the relative frequency histogram, improving significantly the classification performance based on modified features [20], but introducing a bias that results in the estimated probabilities not adding up to a finite number, depending on the time series length *N*. Therefore, these features are no longer an entropy in the sense of the Shannon entropy. However, the term entropy is kept for simplicity since they are still based on that formulation.

From a time series classification perspective, this lack of statistical rigour not only does not produce inaccurate classification results, but rather contributes to unearth additional information, increasing the accuracy of the classification process. Moreover, considering a generic feature set for object classification, there is no need at all for the individual features to satisfy any specific statistical property; only their segmentation power really matters. This is the case for the modified version of PE proposed in [20], and SlpEn [2]. Despite such performance improvement, researchers in the time series entropy realm are more familiar with results within the range 0 and 1, where 0 usually corresponds to completely deterministic series, and 1 to full randomness. In addition, this range is more easily interpretable and dealt with.

Many optimisations methods have been proposed in the scientific literature to improve the performance and robustness of already widely used entropy methods for time series classification. For example, the work [26] describes how to use entropy profiling to improve short length time series analysis using KS–entropy measures, very dependent on input parameter values. They removed the need of manual *r* parameter selection almost entirely. In [27], the authors investigated normalised first order differences of time series to obtain more robust estimates of signal complexity under diverse conditions. Other studies, as [28], illustrate the possible negative effects of normalisation on method performance that should be also taken into account, and are avoided in the present work.

There are also many applicable normalisation schemes described in the literature to scale back any numerical results to the above mentioned [0,1] range [29,30,31], and to reduce the influence of time series length. This is also the case in [32], where the authors apply normalisation to Approximate Entropy to reduce the possible influence on calculations of different lengths.

Along this line, we propose in this work a specific normalisation method for SlpEn to keep its results in the [0,1] range, and make them less dependent on the length of the time series. This method will be based on a–priori estimations of the real number of unique patterns likely to be found, without any detrimental effect on classification performance, and using an approach similar to that in [33] applied to Lempel–Ziv complexity normalisation.

The main contribution of this paper is to propose simple exact and approximate values on which base SlpEn boundaries for normalisation. The practical implications of the study will be illustrated by means of a classification analysis using the SlpEn customisation proposed on both synthetic and real time series of different lengths and properties.

## 2. Materials and Methods

### 2.1. SlpEn

The first step of the SlpEn computation is the extraction from an input time series $x=\left\{x_{0},x_{1},\ldots ,x_{N-1}\right\}$ of a set of consecutive overlapping subsequences of length *m*, commencing at sample *i*, $x_{i}=\left\{x_{i},x_{i+1},\ldots ,x_{i+m-1}\right\}$, 0≤i<N−m+1 (being *m* the embedded dimension variable, and *N* the total length of the time series, with m<<N). Each one of the N−(m−1) extracted subsequences, $x_{i}$, can then be transformed into a new one of length m−1 by computing and storing the differences between each pair of consecutive samples in the subsequence instead, namely, $y_{i}=\left\{x_{i}-x_{i+1},x_{i+1}-x_{i+2},\ldots ,x_{i+m-2}-x_{i+m-1}\right\}$.

Using, in its basic configuration [2], 5 different symbols from a numeric alphabet, for example $S=\left(+2,+1,0,-1,-2\right)$, the differences obtained are represented by these symbols, according to two input thresholds, δ and γ, and the expressions described in [2]. The number of theoretically possible different (m−1)–tuples or strings over this alphabet is given by $5^{m-1}$. In contrast to other entropy methods, in this case symbol repetitions are allowed within each symbolic tuple. For example, for m=3, the strings that can be created with the alphabet *S* are the following $5^{2}=25$ tuples: $\left(0,0\right)$, $\left(0,+1\right)$, $\left(0,+2\right)$, $\left(0,-1\right)$, $\left(0,-2\right)$, $\left(+1,0\right)$, $\left(+1,+1\right)$, $\left(+1,+2\right)$, $\left(+1,-1\right)$, $\left(+1,-2\right)$, $\left(+2,0\right)$, $\left(+2,+1\right)$, $\left(+2,+2\right)$, $\left(+2,-1\right)$, $\left(+2,-2\right)$, $\left(-1,0\right)$, $\left(-1,+1\right)$, $\left(-1,+2\right)$, $\left(-1,-1\right)$, $\left(-1,-2\right)$, $\left(-2,0\right)$, $\left(-2,+1\right)$, $\left(-2,+2\right)$, $\left(-2,-1\right)$, $\left(-2,-2\right)$.

Being $d=x_{j}-x_{j+1}$ the difference between two consecutive samples in a subsequence $x_{i}$, each symbol is computed from $y_{i}$ as:

- +2, if d>γ.
- +1, if d≤γ and d>δ.
- 0, if $\left|d\right|\leq \delta$.
−1, if d<−δ and d≥−γ.
−2, if d<−γ.

For each match with the list of patterns found up to sample *j*, the frequency counter of the corresponding pattern $c_{i}$ is updated. At the end of the process, when all the N−(m−1) subsequences have been parsed, resulting in a histogram of *k* bins (number of unique patterns found *k*), the Shannon entropy is computed to obtain the final SlpEn value, with $p_{i}=\frac{c_{i}}{k}$:

$$SlpEn=-\sum_{i=0}^{k}p_{i}\log p_{i}.$$

Further details of SlpEn implementation and examples can be found in [2,3,34]. A software library using this method is also described in [5].

### 2.2. Experimental Dataset and Baseline SlpEn Results

The present study will use a varied set of both synthetic and real time series in order to assess the validity of the conclusions reached and the expected goodness of the methods proposed. The length of each time series will be later randomly changed to ensure length non–uniformity in the datasets and quantify SlpEn normalisation classification influence in this case.

The specific members of the synthetic experimental dataset are:

- Random. Two classes were generated using Gaussian (Class 0) or uniform (Class 1) amplitude distributions, with 100 records each, of length 5000 samples. This kind of record is included as a representative of a pure random time series.
- Periodic. This group of records consists of two classes of sinusoids, 100 records of period 100 samples (Class 0), and 100 records of period 500 samples (Class 1), both with random phase. The length of all records is 5000 samples. This kind of record is included as a representative of a pure deterministic time series.

and the real time series datasets are:

- Bonn. This database was collected at the Department of Epileptology, University of Bonn [35], and is a frequent dataset found in many similar research studies [10,21,36,37,38,39]. The length of the records is 4097, using two classes of 100 time series each, corresponding to seizure–free (Class 0, F) and seizure–included (Class 1, S) electroencephalograms (EEGs). This dataset was chosen due to its popularity among the scientific community, and because EEGs are the focus of many entropy–related studies.
- House twenty. Time series of the power consumption at 20 UK homes. The records in this database are also publicly available at www.timeseriesclassification.com (accessed on 29 December 2022), and it corresponds to non–physiological data. There are two classes of 20 records for each one, with 1022 samples per record [40]. Class 0 is the household aggregate usage of electricity and Class 1 corresponds to the tumble dryer and washing machine.

Using these datasets described above, and the standard SlpEn method, we obtained some preliminary SlpEn results listed in Table 1, with 100 random realisations for the synthetic time series (results expressed as average and standard deviation). These SlpEn values will be used for reference purposes later in the experiments, to demonstrate the wide span of results to expect, and also to illustrate the effect of time series randomness on SlpEn, which is similar to many other methods: low values for deterministic time series (average SlpEn = −2589.09 for sinus time series), and higher values correlated with the randomness degree (average SlpEn = 39.81 for random time series).

Table 1 also depicts an effect that will be addressed later: results for deterministic time series are more scattered than results for more unpredictable time series. The SlpEn results are denser in the region corresponding to random time series.

### 2.3. Analytic Normalisation

As stated above, our normalisation strategy is rawly based on that applied to Lempel–Ziv Complexity (LZC) [41] proposed in [33]. In that case, the authors addressed the LZC dependence on time series length by finding analytic expressions for both ends of the complexity spectrum: regular sequences (constant and periodic time series), and random sequences, and using these expressions as the lower and upper bounds of the LZC. If $C_{\mathrm{LZC}}$ is the LZC result using the standard method for a time series or sequence of length *N*, $C_{{\mathrm{LZC}}_{\mathrm{const}}}$ is the LZC lower bound obtained from a constant sequence, and $C_{{\mathrm{LZC}}_{\mathrm{rand}}}$ is the upper bound obtained from a random one, the normalisation scheme proposed was:

(1) $$C_{{\mathrm{LZC}}_{\mathrm{normalised}}}=\frac{C_{\mathrm{LZC}}-C_{{\mathrm{LZC}}_{\mathrm{const}}}}{C_{{\mathrm{LZC}}_{\mathrm{rand}}}-C_{{\mathrm{LZC}}_{\mathrm{const}}}}.$$

This normalisation clearly keeps the value of the LZC within the [0,1] interval, and also makes this measure more robust against length differences [41].

Similarly, the basic idea in this case was to estimate in advance the minimum and maximum SlpEn values possible for a given input parameter configuration, and use those extremes as the normalisation limits for each time series being processed, applying a generic expression such as:

(2) $${\mathrm{SlpEn}}_{\mathrm{normalised}}=\frac{\mathrm{SlpEn}-{\mathrm{SlpEn}}_{\min}}{{\mathrm{SlpEn}}_{\max}-{\mathrm{SlpEn}}_{\min}}.$$

Our approximate scheme is based on the variables summarised in Table 2. All these values correspond to parameters related to the input time series itself, or to the SlpEn algorithm. Only the number of unique subsequences found in the time series *k* is obtained once the SlpEn calculation is completed, and this is the main obstacle that will have to be addressed for SlpEn normalisation, as described in the subsections below.

#### 2.3.1. Minimum SlpEn Value. Lower Bound

Let us consider the specific case of a constant gradient time series of length *N*, $x=\left\{x_{0},x_{0}+\Delta ,x_{0}+2\Delta ,\ldots ,x_{0}+\left(N-1\right)\Delta \right\}$, with Δ∈R. Any subsequence extracted from x will have the form $x_{i}=\left\{x_{0}+i\Delta ,x_{0}+2i\Delta ,\ldots ,x_{0}+\left(i+m-1\right)\Delta \right\}$. The vector of differences between consecutive samples will therefore be $y_{i}=\left\{-\Delta ,-\Delta ,\ldots ,-\Delta \right\}$. Depending on the specific input parameters and Δ values, the symbol from *S* assigned will vary, but the sequence assigned will always be the same. In other words, there is only a unique pattern found in this kind of sequence (k=1), being Δ=0 the specific case of a constant time series. From an entropy perspective, this corresponds to the most deterministic case.

Applying the SlpEn computation to a constant gradient time series, it is obvious that there will only be a single histogram bin, accounting for all the N−(m−1) subsequences in x, and the same symbolic string:

(3) $$SlpEn=-\sum_{i=0}^{}p_{i}\log p_{i}=-\frac{\left(N-(m-1)\right)}{1}\log\frac{\left(N-(m-1)\right)}{1}=-\left(N-\left(m-1\right)\right)\log\left(N-\left(m-1\right)\right).$$

It is important to note that m>2 and N>>m in practice, and therefore this expression results in a negative value that decreases with *N*, having *m* a minor influence since its range of variation is also very small (usually 2<m<10). Therefore, Equation (3) can be further simplified and left as:

(4) $$SlpEn_{\min}=-N\log N.$$

This is the exact analytic SlpEn minimum that could be used for range normalisation (${\mathrm{SlpEn}}_{\min}$ in Equation (2)), and it can be known in advance for all the time series in any experimental dataset since it only depends on input parameters *N* and *m* (if used). It is clearly not possible to find any other SlpEn value for any time series and any parameter configuration lower than this one.

#### 2.3.2. Maximum SlpEn Value. Upper Bound

This case is more difficult than the previous one because the number of unique patterns found *k* is unknown in advance for each record, although certainly k>1 (k=1 corresponds to the previous case of minimum SlpEn). Therefore, an approximate approach will be necessary, keeping in mind that such approximation should not have a detrimental impact on SlpEn computational cost or discriminating power.

In order to maximise the Shannon entropy expression, and therefore find ${\mathrm{SlpEn}}_{\max}$, it is necessary to consider the case when the histogram is uniform, namely, the relative frequencies of all the patterns found are equal. If the number of unique patterns is *k*, and there are N−(m−1)≈N subsequences of length *m* in x, the height of each bin in the histogram is $\frac{N}{k}$. Thus, the general SlpEn expression can be written as:

(5) $$SlpEn=-\sum_{i=0}^{k-1}p_{i}\log p_{i}=-k\frac{\left(\frac{N}{k}\right)}{k}\log\frac{\left(\frac{N}{k}\right)}{k}=\frac{N}{k}\log\frac{k^{2}}{N}.$$

In order to find out the upper limiting value of SlpEn, we have to compute the *k* value that maximises Equation (5). Considering that equation as a function f(k), and obtaining $\frac{df(k)}{dk}=0$, we have:

$$\frac{df(k)}{dk}=\frac{2N}{k^{2}}-\frac{N\log\frac{k^{2}}{N}}{k^{2}}=0,$$

from which $k=e\sqrt{N}$ maximises SlpEn as ${\mathrm{SlpEn}}_{\max}=\frac{2\sqrt{N}}{e}$.

Combining all the previously computed maximum and minimum bounds together, Equation (5) becomes, finally:

(6) $${\mathrm{SlpEn}}_{\mathrm{normalised}}=\frac{\mathrm{SlpEn}-\left(-N\log N\right)}{\frac{2\sqrt{N}}{e}-\left(-N\log N\right)}.$$

### 2.4. Heuristic Normalisation

We have proposed in Section 2.3 simple to compute interval bounds to use for SlpEn normalisation and ensure its results will always be between 0 and 1. These bounds were −NlogN and $\frac{2\sqrt{N}}{e}$. It is certainly not possible to find any time series that results in a SlpEn value out of that interval. However, these bounds were very conservative and, as a consequence, the normalised values of real time series fall within a small region of the $\left[0,1\right]$ interval, making interpretation of the results less convenient.

**Figure 1:**
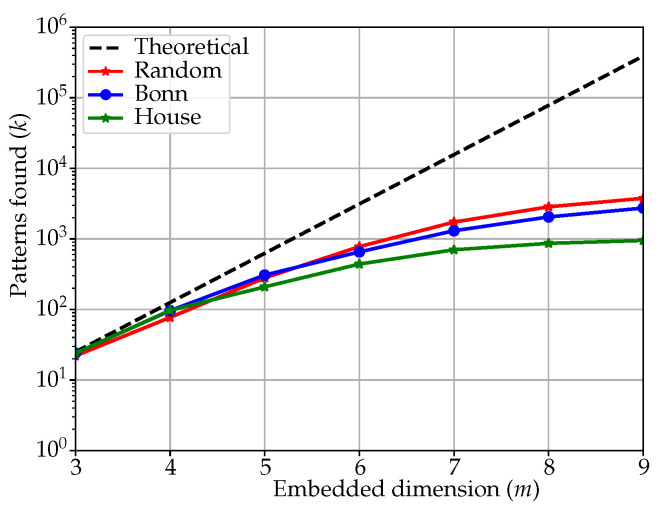
Empirical relation between the theoretical number of unique patterns expected, $5^{m-1}$, and the real number found, *k*. The curves correspond to maximum values obtained during the analysis of all the records in each dataset, and for different input parameter configurations. The maximum *k* is expected to be for random time series, but still lower than the value predicted theoretically.

The main objective of this section is to refine the normalisation process with more practical bounds, achieving a more intuitive SlpEn distribution across the entire $\left[0,1\right]$ interval. Specifically, the previous approach used was too conservative because:

- Regarding the lower bound, it corresponds to a constant time series, or, similarly, to a constant slope time series, which are of no interest in real entropy analysis applications normally. This lower bound grows very rapidly when adding even minor variability to the records. For example, when a random change is introduced in a single value of a constant time series (a new pattern will be found), there is an abrupt shift in the entropy result. If more changes were added, this shift becomes more significant, even if the time series is still mostly constant. The mathematical expression that illustrates this effect when a single match is added to an otherwise constant slope time series is shown below:
  (7) $${\mathrm{SlpEn}}_{\mathrm{constant}}=-\frac{N-\left(k-1\right)}{k}\log\frac{N-\left(k-1\right)}{k}-\left(k-1\right)\frac{1}{k}\log\frac{1}{k}.$$
  When the number of patterns found *k* is 1, this is the case of the minimum SlpEn value (Equation (3)). Then, we can consider the case in which the time series is still mainly constant, but with scattered disturbances that can subtract a pattern from the dominant case and create a new histogram bin with a single pattern (although a single outlier in the time series might result in more than one new symbolic pattern, depending on *m* value). As a result, we can quantify how the SlpEn of a pure constant slope time series evolves when new patterns arise, k=2,3,4,…, and the amplitude of the dominant bin decreases accordingly, with a single match per pattern (Equation (7)).
  In order to illustrate the numerical influence of this effect, Table 3 shows the SlpEn results of a 5000 samples constant gradient time series to which a new pattern and a single match is added, from 2 up to 10, regardless of the input parameters’ values. The minimum SlpEn value for this time series is −61,369.91, but still mostly constant slope time series yield a SlpEn of −4465.38 and beyond. In other words, the interval [−61,369,−4465] is infra–utilised in terms of normalisation, the ${\mathrm{SlpEn}}_{\min}$ bound could be shifted towards greater values without losing interpretability. This is also visually illustrated in Table 3.
  The case of a pure periodic record is also very illustrative in this same regard. Periodic time series are also periodic in terms of symbolic patterns, that is, once a complete period has been parsed, the distribution of the histogram remains the same with the addition of new periods (depending on series total length). Although its SlpEn result will be greater than that of a completely constant record, since a few patterns have most of the impact on the SlpEn result, their ${\mathrm{SlpEn}}_{\min}$ is still far smaller than that of a real time series, since periodic records are also completely deterministic. This is numerically illustrated in Table 4.
  As a consequence, the interval between the analytical ${\mathrm{SlpEn}}_{\min}$ and the point where SlpEn of real time series lies, consumes a large part of the entire [0,1] interval, not a fair representation of the determinism–randomness balance found in real signals, which are of greater practical interest and the focus of entropy analyses. Therefore, ${\mathrm{SlpEn}}_{\min}$ could be increased to leave deterministic time series below that threshold and assign them an entropy value of 0 directly.
- Regarding the upper bound, $\frac{2\sqrt{N}}{e}$, it can also be very conservative since it derives from the maximisation of the analytical expression of SlpEn when the histogram is uniform, which is extremely unlikely to achieve in a real case. Since there is no prior knowledge about this value, an analytic study is not optimal, and the upper bound should be based on heuristics and approximations instead.
  In order to characterize the behaviour of *k* in relation to *m* in SlpEn (N>>m), we conducted a preliminary analysis using some of the records described in the experimental dataset. The results of this analysis are shown in Figure 1, with a comparison between the theoretical number of patterns expected to be found in random and real time series, and the actual number found *k*, which is clearly and significantly smaller.
  Figure 1 includes a few examples of what also happens with many more other time series and entropy measures: the number of different patterns found is several orders of magnitude smaller than what theory predicts. This was also illustrated for PE in the [20] study. Taking advantage of this difference by means of a robust estimation, the upper bound could be refined with a more realistic approximation of *k*.

#### 2.4.1. Refinement of the Lower Bound

The SlpEn of real time series is mainly located at the high end of the $\left[0,1\right]$ interval. At the opposite end of the range there is a relatively wide subrange of results for constant, periodic, or highly deterministic time series, not usually found in real life applications. For example, results in Table 3 show that SlpEn values for deterministic time series range from −60,000 up to −4000 and beyond, whereas for more unpredictable ones it ranges from −6 up to 40 approximately (Table 1). There is a clear unbalance between the range covered by determinism and randomness, which has a detrimental effect on the analytical normalisation proposed in Section 2.3. In other words, most of the range for normalisation is devoted to time series that are not the focus of real life analyses. This can be improved using a logarithmic normalisation instead of a linear one, but only improves the results’ region of the deterministic time series.

**Figure 2:**
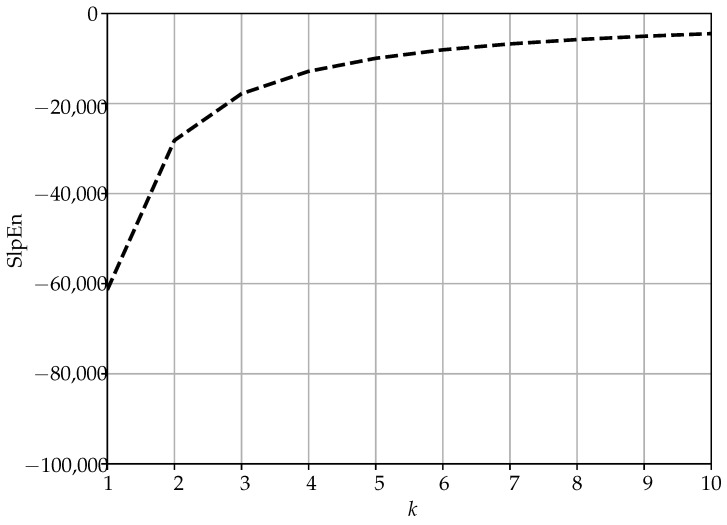
Influence of the addition of new patterns on SlpEn based on results in Table 3 for a constant slope time series.

The practical consequence of this is that the proposed interval $\left[-N\log N,\frac{2\sqrt{N}}{e}\right]$ can be far too wide both for linear or logarithmic normalisations. It does not make much sense to calculate an entropy measure of a constant or constant gradient time series since they are not found in real life contexts. This is also the case for pure periodic series, with an infinite signal to noise ratio, that is, the number of patterns found is very low and constant regardless of the time series length (Table 4). Moreover, even in a constant time series, a variation of a single sample (due to noise for example) that results in a different symbol, has a significant impact on the histogram distribution, and the calculation of the Shannon entropy (Figure 2).

For example, let $x=\left\{1.0,1.0,\ldots ,1.0\right\}$ be a constant time series of length *N*. It is clear all the subsequences in x result in the same SlpEn symbolic string, (0,0), when m=3. Therefore, its SlpEn value is exactly $\left(-N+(m-1)\right)\log\left(N-(m-1)\right)$, since k=1, as justified in Section 2.3. If now the time series is very slightly modified, resulting in, for example, $x=\left\{1.0,1.0,\ldots ,2.0,1.0,\ldots ,1.0\right\}$ (a single different value that results in a different symbol if $\delta =1\cdot 10^{-3}$). It is clear that the number of unique patterns is increased to m+1, the predominant pattern, and one additional new pattern for each position the outlying value takes in a subsequence, *m* different locations (provided the outlier is not at the borders of the time series, for a generic case).

In order to illustrate this effect numerically, let us consider a constant time series with N=66, δ=1e−3, γ=0.2 and m=4. In this case, its original SlpEn result is −376.57. Adding an outlier that results in adding *m*, that is, 4 additional patterns, SlpEn becomes −40.16. Under the same conditions, SlpEn for a random time series is 6.97. For a pure periodic time series (T=3), SlpEn is −58.95. In other words, the logarithmic nature of the entropy measure makes SlpEn to use a major part of the $\left[0,1\right]$ interval to account for time series that are of little interest in this kind of analyses. The lower bound could therefore be raised to leave these cases out (results below the new bound saturated towards 0), and optimise the interval to better represent real time series. Following the previous example, the lower interval limit could have been safely shifted from −376.57 to −40.16, assigning a SlpEn value of 0 for the constant and periodic time series and contribute to a better distribution of values between 0 and 1 for real cases.

Based on this reasoning, and using the values obtained in Table 1, Table 3 and Table 4, it was concluded that a safe approximation for the lower bound was to consider the SlpEn result that corresponds to that of Equation (7) when a new pattern is added for each 100 samples in the time series. Obviously this is an heuristic approximation that could be chosen differently depending on the application and user needs and preferences, provided deterministic time series fall below the new 0 SlpEn threshold, but not real ones.

#### 2.4.2. Refinement of the Upper Bound

In this case, the main obstacle to find a simple boundary is that *k* is not known until SlpEn is computed, and therefore the real ${\mathrm{SlpEn}}_{\max}$ can not be obtained. If at least a reasonable preliminary estimation of *k* could be computed, that would certainly contribute to fine tune the normalisation interval.

When the number of symbols and how they can be arranged is known, the quantity of all different combinations or permutations that can be found theoretically is easily computed. This is the case of PE, and also of SlpEn, with $5^{m-1}$, as stated in Table 2.

Other methods use this theoretical number of patterns expected, and therefore the normalisation is more straightforward. However, as previously stated, the effect of forbidden patterns or the differences in pattern probability are very good markers of time series properties, with a high discriminating power, and that approach keeps statistical rigour but at the expense of classification accuracy and robustness. Therefore, that is not an option in this case.

In a real scenario, as illustrated in Figure 1, the number of possible combinations, and the actual number found, can be very different. This can be due to two reasons: the length of the time series is not enough to allocate all the pattern variations, especially when some of them have a low appearance probability, or due to the concept of forbidden patterns [25,31,42]. In any case, the real number of unique strings has been demonstrated to be a marker of the randomness degree of the input time series [24], and be a powerful distinguishing feature for classification [20] that should not be overlooked.

In order to estimate in advance the *k* value sought, we studied the results in Plot Figure 1, specifically the case of random time series, the most restrictive one. Those real *k* values are shown in Table 5. The *k* value for which the maximum SlpEn is analytically achieved is also included.

From Table 5, two practical consequences could be derived. First, as already stated, the number of patterns found is smaller than that expected, and therefore the maximum SlpEn is not achieved when the histogram is uniform, and there are many missing patterns. Second, in some cases, *k* does not reach the value needed to maximise SlpEn (in Table 5, for m=3 and m=4), depending on *N*. Consequently, *k* should be bounded by the maximum theoretically possible patterns, not just *N*, and a heuristic estimation of *k* could be devised from the results obtained using random time series.

This relationship between the theoretically possible different patterns in a time series, $5^{m-1}$, and the unique patterns actually found, *k*, as a function of *m*, was approximated using several regression models. From a varied and diverse set of possible functions, those with a correlation coefficient greater than 0.9 were:

- Linear regression, y=650.4643x−2546.2143, with a correlation coefficient of 0.9522.
- Quadratic regression, $y=116.5119x^{2}-747.6786x+1182.1667$, with a correlation coefficient of 0.9970.
- Cubic regression, $y=-13.3056x^{3}+356.0119x^{2}-2091.5397x+3497.3333$, with a correlation coefficient of 0.9985.
- Power regression, $y=0.0998x^{4.9136}$, with a correlation coefficient of 0.9464, and the smallest relative error, 12%.

Therefore, *k* can also be estimated as only dependent of *m*, using the relationship found $k=0.0998m^{4.9136}$, or similar. The calculation of $SlpEn_{\max}$ can use this new value of *k* instead of $e\sqrt{N}$, resulting in a the new term that can be applied to Equation (2) to complete the normalisation process. However, as will be illustrated in Section 3, the initial upper bound of $\frac{2\sqrt{N}}{e}$ offered good results already, close to 1, and better distributed than for deterministic time series. This refinement is less necessary than that for the lower bound described in Section 2.4.1.

## 3. Experiments and Results

### 3.1. Results Using the Analytic Bounds

Table 6 shows the SlpEn results (mean ± standard deviation) for each type of record in the experimental dataset using the initial normalisation bounds described in Section 2.3. The first row corresponds to the analytic normalisation process described in the present study, and the second one to the standard SlpEn method without normalisation. As can be observed in the results in each case for classification sensitivity and specificity (Class 0 is the positive class), the normalisation process does not impact on the classification performance of SlpEn.

Table 7 and Table 8 show also the results for the normalised and standard SlpEn methods, but in this case, the time series length of each record was varied. These experiments were devised to study the length influence on both SlpEn algorithms. Only the results for Random and Bonn datasets are provided.

### 3.2. Results Using the Heuristic Bounds

Table 9 shows the SlpEn results (mean ± standard deviation) using the heuristic bounds based on approximations and described in Section 2.4. In this case, there are greater differences among the results for each group. The classification performance remains the same except for the Periodic database, as discussed later. The length analysis was not carried out in this case, since the behaviour of the results was exactly the same as in Table 7 and Table 8.

However, although the heuristic minimum bound seems to have a great impact on the normalisation process, the heuristic maximum does not have the same impact, and it could be discarded for further simplification. This is justified in Table 10, where the results were obtained using the analytic maximum and the heuristic minimum bounds for normalisation. The results are not as close together as in Table 7, and there is no risk of having a combination of input parameters and time series exceeding the 1 value since the maximum bound is not based on approximations. Moreover, any regression analysis to obtain a heuristic relationship may be too costly for the possible benefit achieved.

### 3.3. Results Using Very Short Records

Since one of the main goals of this normalisation study was to ensure the classification accuracy was not impacted negatively, the experiments in this section were devised to analyse this accuracy under more difficult conditions than in previous experiments, specifically, using very short records. The results achieved are shown in Table 11 for real datasets Bonn and House, and for a number of samples from 100 up to 500 in 100 steps.

### 3.4. Results Using Undersampling

In the previous subsection, we studied the possible influence of using shorter versions of the input time series. In this case, the experimental dataset used is also shorter, but due to downsampling instead. The results are again the same between the two methods employed. These results are shown in Table 12.

## 4. Discussion

Results in Table 6, Table 7, Table 8, Table 9 and Table 10 show normalised SlpEn values remain between 0 and 1, and its classification performance is unaffected; regardless, the normalisation method was used, which were the main goals of the present study.

The comparison in Table 6 using the analytic bounds for normalisation, $\left[-N\log N,\frac{2\sqrt{N}}{e}\right]$, confirmed the sensitivity and the specificity of both SlpEn variants were exactly the same. The minimum normalised SlpEn value was achieved by the most deterministic data set, the periodic, with values in the vicinity of 0.95±0.01. On the opposite end, the random dataset achieved the maximum normalised value, around 0.99. Although this range falls within the goal of [0,1], it is probably too narrow to provide a good perspective of the randomness of the datasets, mainly at the minimum level.

That is why heuristic bounds better matching real entropy analysis schemes were devised and applied. The corresponding results were shown in Table 9. In this case, the results were 0.00 for the Periodic data set, and 0.99 for the House dataset. The differences were mainly due to the new normalisation scheme, but the specific case of the Periodic database, with 0.00 SlpEn, was due to the thresholding applied, with 0 SlpEn for those time series considered deterministic. Obviously, this results in a 0% classification accuracy for the Periodic database. If that is not acceptable, the threshold can be customised depending on the application.

However, comparing Table 6 and Table 9, it becomes apparent that the maximum heuristic bound is not really necessary, since the logarithmic nature of the expressions expands mainly the lower part of the interval. This is justified by the results in Table 10, where using the minimum heuristic bound, and the exact analytic maximum, the results are still reasonably distributed according to their determinism degree. Therefore, this seems to be the most efficient solution for the objective of the present paper.

Finally, in order to illustrate the effect of length on standard SlpEn calculation, more experiments were conducted varying the length of records from 1000 up to 4000 samples, in 1000 samples steps, and the results reported in Table 7 and Table 8 (only for Random and Bonn databases). As can be observed in such Tables, the normalisation method devised also reduces the SlpEn dependence on time series length. On this same matter, results in Table 11 show the performance of the normalisation method in its final recommended version is also equal to that of the original one even under the difficult conditions that very short records entail, as with the downsampling shown in Table 12.

## 5. Conclusions

This paper proposes to use a Max–Min normalisation scheme to keep the results of SlpEn within the interval [0,1]. The main difficulty to apply any normalisation to SlpEn is that some parameters are not known until the record is processed; in this case, the number of unique patterns found *k*, from which the maximum value of SlpEn can be derived.

The first approach proposed uses an analytic technique that computes the limits from assumptions about the minimum possible SlpEn value, which coincides with the SlpEn of a constant gradient time series (−NlogN), and the maximum SlpEn value, based on a uniform histogram and maximisation values for *k* ($\frac{2\sqrt{N}}{e}$). This approach is easily implemented, keeps SlpEn within the desired interval, and do not damage the classification performance. The main weakness of this approach is that SlpEn results are too close to 0.9–1.0, and differences are not visually very apparent.

The second approach shifts the bounds to values based on real cases, when very deterministic time series such as constant or periodic records are of no interest in entropy terms, and therefore it is not necessary to keep a part of the interval for them, just the 0 value. On the opposite bound, it can be shown empirically that the number of patterns found is usually several orders of magnitude smaller than that theoretically expected, and this relationship can be estimated and applied to refine the upper bound.

Using this last approach, the SlpEn results are better distributed. However, with a global analysis of all the results, it seems the optimal combination of bounds is the minimum heuristic bound $-\frac{N-\left(k-1\right)}{k}\log\frac{N-\left(k-1\right)}{k}-\left(k-1\right)\frac{1}{k}\log\frac{1}{k}$, and the analytic maximum, given by $\frac{2\sqrt{N}}{e}$. These are the final bounds recommended to be included in the computation of SlpEn to obtain a method with less disparity in the result values.

In future studies, this kind of normalisation or a derived one could be also customised to make SlpEn almost independent of *N* to improve accuracy when applied to non–uniform datasets in terms of length. That is the case of records such as body temperature or blood pressure records [43,44], where each time series frequently has a different length due to many acquisition artifacts [11]. Other length reduction techniques such as trace segmentation [45] should be assessed.

## Figures and Tables

**Table 1:** Illustrative SlpEn results for the experimental sets. The input parameters were m=6, δ=0.001, γ=0.8. It is important to note the good correlation between the randomness of the input time series and the SlpEn result, and the greater span for the deterministic or relatively deterministic records. As stated in the text, negative values are possible since relative frequency normalisation only considers the number of times a pattern really occurred, not all the possible outcomes.

	Random	Periodic	Bonn	House
Class 0	39.83±0.35	−3422.90±375.63	18.95±11.09	19.46±1.84
Class 1	39.29±0.38	−1739.62±0.00	−30.40±64.41	20.78±1.04

**Table 2:** Summary of the variables involved in the approximate scheme described for SlpEn normalisation.

Parameter	Value
Total length of the time series x	*N*
Length of the subsequences (embedded dimension)	*m*
Total number of subsequences to extract in x	N−(m−1)
Length of the symbolic subsequences in SlpEn	m−1
Length of the symbol alphabet	5, symbols +2, +1, 0, −1 and −2
Theoretical maximum number of unique subsequences that can be found	m−1 tuples over 5 elements, $5^{m-1}$
Real number of unique subsequences found	*k*, only known after SlpEn computation

**Table 3:** Numerical impact of the addition of new patterns to the SlpEn of a constant slope time series. Due to the logarithmic nature of the calculations, the addition of just 0.2% unique patterns, entails a 13.75 times SlpEn variation, despite remaining the time series to be mostly constant.

*k*	1	2	3	4	5	6	7	8	9	10
SlpEn	−61,369.91	−28,180.74	−17,808.36	−12,835.14	−9943.67	−8064.59	−6751.82	−5786.18	−5048.07	−4465.38

**Table 4:** SlpEn results for a periodic time series of length 5000 using different period lengths. Embedded dimension *m* was 6 in all cases. The number of patterns found was between 20 and 35.

Period	10	9	8	7	6	5	100	1000	5000
SlpEn	−8278.22	−8172.91	−8058.11	−5583.10	−5836.22	−6081.71	−7316.64	−4641.32	−8917.78

**Table 5:** Numerical relationship between the expected number of unique patterns to be found $5^{m-1}$, and the maximum actual number found *k* in a set of 100 random time series of length 5000. The *k* value for which the maximum SlpEn is analytically achieved is also included.

*m*	3	4	5	6	7	8	9
$5^{m-1}$	25	125	625	3125	15,625	78,125	390,625
max(*k*) found	22	77	280	777	1730	2847	3763
$e\sqrt{N}$	192	192	192	192	192	192	192

**Table 6:** Results using the analytic bounds for SlpEn normalisation. Average results including all classes. Input parameters were m=6, γ=0.8, and δ=0.001.

		Random	Periodic	Bonn	House
${\mathrm{SlpEn}}_{\mathrm{normalised}}$	Result	0.99942±0.00	0.95669±0.01	0.99850±0.00	0.99861±0.00
	Sensitivity	0.83±0.18	1±0.00	0.81	0.70
	Specificity	0.85±0.15	1±0.00	0.77	0.75
SlpEn	Result	39.81024±0.42	−2589.09±890.76	−5.72605±55.41	20.12±1.62
	Sensitivity	0.83±0.18	1±0.00	0.81	0.70
	Specificity	0.85±0.15	1±0.00	0.77	0.75

**Table 7:** Average results for Random database and different lengths. Input parameters were m=6, γ=0.8, and δ=0.001.

		1000	2000	3000	4000
${\mathrm{SlpEn}}_{\mathrm{normalised}}$	Result	0.99822±0.00	0.99885±0.00	0.99914±0.00	0.99931±0.00
	Sensitivity	0.72±0.24	0.75±0.14	0.68±0.27	0.93±0.11
	Specificity	0.89±0.19	0.88±0.13	0.82±0.28	0.66±0.04
SlpEn	Result	15.77054±0.25	22.40642±0.21	28.50014±0.39	34.25239±0.28
	Sensitivity	0.72±0.24	0.75±0.14	0.68±0.27	0.93±0.11
	Specificity	0.89±0.19	0.88±0.13	0.82±0.28	0.66±0.04

**Table 8:** Average results for Bonn database and different lengths. Input parameters were m=6, γ=0.8, and δ=0.001.

		1000	2000	3000	4000
${\mathrm{SlpEn}}_{\mathrm{normalised}}$	Result	0.99754±0.00	0.99800±0.00	0.99830±0.00	0.99849±0.00
	Sensitivity	0.80	0.73	0.74	0.82
	Specificity	0.71	0.85	0.86	0.77
SlpEn	Result	9.00236±21.96	3.67452±44.43	−0.56024±46.20	−5.04276±53.78
	Sensitivity	0.80	0.73	0.74	0.82
	Specificity	0.71	0.85	0.86	0.77

**Table 9:** Results using the heuristic bounds for SlpEn normalisation. Average results including all classes. Input parameters were m=6, γ=0.8, and δ=0.001.

		Random	Periodic	Bonn	House
${\mathrm{SlpEn}}_{\mathrm{normalised}}$	Result	0.92275±0.00	0.00000±0.00	0.88183±0.07	0.99021±0.00
	Sensitivity	0.63±0.08	0.00±0.00	0.81	0.70
	Specificity	0.79±0.04	0.00±0.00	0.77	0.75
SlpEn	Result	39.67282±0.53	−2589.09765±890.76	−5.72605±55.41	20.12±1.62
	Sensitivity	0.63±0.08	1±0.00	0.81	0.70
	Specificity	0.79±0.04	1±0.00	0.77	0.75

**Table 10:** Results using the heuristic minimum bound and the analytic maximum bound for SlpEn normalisation. Average results including all classes. Input parameters were m=6, γ=0.8, and δ=0.001.

		Random	Periodic	Bonn	House
${\mathrm{SlpEn}}_{\mathrm{normalised}}$	Result	0.95095±0.00	0.00000±0.00	0.90009±0.07	0.98029±0.02

**Table 11:** Classification results (Sensitivity/Specificity) for Bonn and House databases using short epochs at the beginning of the original epochs. Length ranges from 100 up to 500 samples in 100 steps. Input parameters were optimised to maximise accuracy.

	Samples:	100	200	300	400	500
Bonn	SlpEn	0.89/0.85	0.95/0.88	0.91/0.90	0.92/0.92	0.90/0.95
	${\mathrm{SlpEn}}_{\mathrm{normalised}}$	0.89/0.85	0.95/0.88	0.91/0.90	0.92/0.92	0.90/0.95
House	SlpEn	0.80/0.60	0.70/0.95	0.90/0.90	0.90/1	1/0.90
	${\mathrm{SlpEn}}_{\mathrm{normalised}}$	0.80/0.60	0.70/0.95	0.90/0.90	0.90/1	1/0.90

**Table 12:** Classification results (Sensitivity/Specificity) for Bonn and House databases using short epochs due to downsampling. Downsample rate ranges from 2 up to 5 (take 1 out of 5 samples). Input parameters were optimised to maximise accuracy.

	Decimation:	2	3	4	5
Bonn	SlpEn	0.85/0.97	0.90/0.89	0.92/0.86	0.93/0.84
	${\mathrm{SlpEn}}_{\mathrm{normalised}}$	0.85/0.97	0.90/0.89	0.92/0.86	0.93/0.84
House	SlpEn	0.85/0.95	1/0.80	1/0.90	0.95/0.90
	${\mathrm{SlpEn}}_{\mathrm{normalised}}$	0.85/0.95	1/0.80	1/0.90	0.95/0.90
